# Prevalence and patterns of premenstrual disorders and possible association with sexual harassment: a cross-sectional study of young Arab women

**DOI:** 10.1186/s12905-022-02130-0

**Published:** 2022-12-21

**Authors:** Yossef Hassan AbdelQadir, Ahmed Assar, Yomna Ali Abdelghafar, Manar Ahmed Kamal, Mohamed Sayed Zaazouee, Sarah Makram Elsayed, Khaled Mohamed Ragab, Ayman Essa Nabhan, Nahla Ahmed Gamaleldin, IMedRA collaborators team, Mariam Salah Moris, Batool Emad AL-Masri, Dina M. El-Sherif, Yomna Mohamed Goudy, Asmaa Khaled Alsiouty, Maysa Madny Mahmoud, Hazem Metwally Faragalla, Ebtehal Ahmed Abdelazim, Nadine Abdel-Hamied Mohamed, Sarah Ibrahim Elsayed, Hala Hassan Shehata, Sara Mohamed Hasan, Rana Hanafy Mahmoud, Israa Ashraf Fathy, Eman Mohamed Gomaa, Sara Abdallah Ezz, Dana Alqatawneh, Yasmin Salah Hrezat, Kawther Saleem Alhefnawi, Lina Omar Hasan, Hadeel Naem Saleh, Ayah AbdElWaley Abed, Tharwah Mohammad Rashed, Nedaa Yousef Ahmad Ikhlaif, Rand Adnan Bashir, Sallam Mohammad Alrosan, Merana khalil Ayyoub, Muna Mowafaq Labeeb, Orjuwan Omar AbuShanab, Noor Y. Fraihat, Haya Al Jabban, Amina Ahdab, Homam Alolabi, Sandy Adel Al Khalil, Mohamad klib, Wisam Azzouz, Inas A. Haza’a Allazkani, Lana Talal Wannes, Maya Abdullah Naem, Nada M. Molham Al Barudi, Shatha Alassi, Joudi Saadeddin Tarabishi, Rami Anadani, Hasan M. Masoum Hamoud

**Affiliations:** 1 Faculty of Medicine, Alexandria University, Alexandria, Egypt; 2 Faculty of Medicine, Menoufia University, Menoufia, Egypt; 3 Faculty of Medicine, Benha University, Benha, Egypt; 4 Faculty of Medicine, Al-Azhar University, Assiut, Egypt; 5 Faculty of Medicine, October 6 University, Giza, Egypt; 6 Faculty of Medicine, Minia University, Minia, Egypt; 7 Faculty of Medicine, Al Andalus University for Medical Sciences, Tartus, Syria; 8 Lecturer of Public Health and Community Medicine, Faculty of Medicine, Alexandria University, Alexandria, Egypt; 9 International Medical Research Association (IMedRA), Cairo, Egypt

**Keywords:** Premenstrual dysphoric disorder, Premenstrual syndrome, Prevalence, Sexual harassment, Young women

## Abstract

**Background:**

Premenstrual syndrome (PMS) and premenstrual dysphoric disorder (PMDD) represent a range of both psychiatric and physical symptoms that impair quality of life and interfere with daily activities in females.

**Aims:**

To assess the prevalence of PMS and PMDD in Egypt, Jordan and Syria, its demographic associations and the potential link to sexual harassment (SH).

**Methods:**

We used an Arabic version of the premenstrual symptoms screening tool (PSST) to assess the prevalence of PMS and PMDD. Another two-part questionnaire was used to assess the harassment experience.

**Results:**

22,021 women agreed to fill the questionnaire; the majority (65%) aged 18–25 years old. PMS was more prevalent in Egyptian women 77.7% followed by women from Jordan 72.9% then Syria 66.3%. PMDD prevalence followed the same order (40%, 34.7% and 28.2%). Both conditions were significantly associated with obesity and working in medical careers (*P* = .001). 5733 women agreed to share their sexual harassment experience. Results showed a significant association between the diversity and frequency of sexual harassment and the frequency of the pre-menstrual conditions, PMS Frequency was 87.6%, 80.7% and 78% in participants who were harassed on daily basis Vs. once weekly or monthly vs. few times in their lifetime (*p* = .04). A similar statistically significant difference was noticed regarding having PMDD (66.4% vs. 47.6% vs. 42.5%).

**Conclusion:**

The study showed high levels of both PMS and PMDD. The data provided by this study also sheds light on a potential link between SH and developing Pre-menstrual disorders.

**Supplementary Information:**

The online version contains supplementary material available at 10.1186/s12905-022-02130-0.

## Introduction

Premenstrual syndrome (PMS) is a group of psychiatric and physical symptoms that occur during women's reproductive period mostly on a cyclic basis, with a worldwide mean prevalence of 47.8% [1]. It starts within the late luteal phase of the menstrual cycle and continues till the first few days after menstruation for around six days [2]. It has a negative impact on women's functional, social, and occupational life [3]. Also, Lower scores on Work-related quality of life Scale were observed in PMS patients [4].

Premenstrual dysphoric disorder (PMDD) is defined as a constellation of mood, behavioral, and somatic symptoms that is included in the diagnostic and statistical manual of mental disorders 5th edition (DSM-5) as one of the depressive disorders, which require 4 criteria for diagnosis [5]. It has some established comorbidities such as, anxiety disorders and major depression [6]. Women with PMS and PMDD may experience a variety of different mental and physical symptoms such as mood instability, anxiety, increased appetite, back pain, breast tenderness and bloating [2]. It is a multifactorial condition, as some studies propose hormonal, genetic and environmental factors, but the exact mechanism is not clear [7].

Despite the serious burden that this disease has on women’s lives, there is little data from the Arab countries in the Middle East about its actual prevalence and associated factors, this is owing to the conservative nature of these communities, and the taboo this disorder and other menstrual disorders and sexual concepts represent in people’s cultures which make it harder for women to discuss this gender-related conditions in public.

Sexual harassment is one of these big taboos that is dramatically underreported although it is very prevalent among adolescents in the middle east [8]. It is defined as an unwelcome sexual advances in the form of verbal or physical nature either implicitly or explicitly [9]. Recent studies have linked sexual harassment with many psychological disorders including depression, anxiety, and posttraumatic stress disorder (PTSD) [10]. Certain physiological changes occur with sexually harassed women that can account for these diseases. In a study by Lemieux et al., they discovered that females diagnosed with PTSD after sexual abuse have chronic elevation of neuroendocrine mediators such as norepinephrine and cortisol [11]. Also, Girdler et al. found a neuroendocrine dysfunction and sympathetic hyperactivity in women who had PMDD after prior exposure to sexual abuse [12]. This indicates that women with history of sexual abuse have aggravated response to mental stressors, and hence increase PMS and PMDD. Many observational studies reported an association between history of sexual harassment and diagnosis of PMS or PMDD in different populations [13–16]. However, data are lacking from the Arab counties despite being notorious for having a relatively high rate of sexual abuse.

In Egypt, the limited data on PMS prevalence are collected from small groups of students or nurses, which can't be generalized to the population. For example, the prevalence is estimated to be 51.5% among secondary school girls [17]. Prevalence is also reported in individual governorates as (56.1%) [18] and (86.3%) [19]. Numbers are not widely different in Jordan as it is reported to be 80.2% in one Jordanian college [20], and 92.3% among college girls in Jordan as a whole [21]. There is no data regarding prevalence from Syria. As observed, most of these results from the individual studies are limited to very specific group sample and are not representative of the country's burden of the disease.

So, in this study, we aim primarily to estimate the prevalence of pre-menstrual disorders in Egypt, Jordan and Syria, in addition to its association with different demographic factors. As a secondary objective, we aim to investigate the possible association between prior exposure to major event such as sexual harassment and diagnosis of PMS and PMDD.

## Methods

### Study design, aim and hypothesis

This study was designed as an online, survey-based cross-sectional study that aims to assess the prevalence and the demographic pattern of pre-menstrual syndrome (PMS) and pre-menstrual dysphoric disorder (PMDD) among young females in three Arab countries. Also, this study aims to explore the potential association with sexual harassment.

### Presentation of the hypothesis

In addition to the intended estimation of the prevalence of pre-menstrual disorders in the study sample, this study hypothesized the presence of a demographic pattern in the frequency of pre-menstrual disorders with some demographic groups expected to show higher frequency than others. As a secondary objective, this study also hypothesized that an association exists between pre-menstrual disorders and sexual harassment based on previous observations in the literature as discussed earlier. Relevant data was collected and appropriate statistical tests were implied to test these hypotheses.

### Setting

The study was conducted via an online questionnaire distributed on social media platforms (Facebook, Twitter, and linked-in), targeting participants from Egypt, Jordan, and Syria. The data collection was done from 1st November to 14th November 2020.

### Participants

#### Inclusion criteria

Females aged 18–35 from Egypt, Jordan, and Syria were invited to participate in the study by filling out the questionnaire.

#### Exclusion criteria

Responses from those aged below 18 or above 35, those from outside the three included countries, and those diagnosed with a mental health condition or a hormonal disorder or those on hormonal birth control were excluded before the analysis. Responses with missing data were also excluded.

### Instruments used to measure the variables of the study

We used a three-part questionnaire to measure the variables in the study.

- *First part* aimed to assess the demographics of the study participants and included questions about (age, country, height, weight, marital status, academic degree, job, number of work hours, and whether or not they were previously diagnosed with a mental health condition or a hormonal disorder). For those who were ever married, questions about hormonal birth control, number of pregnancies, and number of births were added.
- *Second part* Aimed to assess the prevalence of PMS and PMDD. We used the “Pre-menstrual symptoms screening tool (PSST)” [22]. The tool assesses PMS and PMDD by asking about 14 different symptoms and five different life domains with which these symptoms might interfere. The screening tool was translated from English to Arabic by two independent translators, then backward translation of the Arabic version to English was done by a third translator so that a final translated version could be agreed upon. The translated version was piloted on 20 women from each country for clarity. Validity and internal consistency of an Arabic culturally adapted version of the screening tool was reported previously in the literature [24], internal consistency of the questions in this current study was 0.86.
- *Third part* aimed to assess the harassment experience in the participating women. First, the respondents were directed to another page to explain and define the meaning of sexual harassment. Then, the women would be asked if they have had such experience that matches the description and if they are willing to share it with the investigators for scientific purposes. The women who agreed to share their experience would be directed to a questionnaire that consisted of two parts:

1. *The first part* aimed to quantify the diversity of the harassment experience. In this part, women were asked yes/no questions to assess if they experienced 13 different acts of harassment (Chronbach's alpha = 0.7) as follows:

- *Two types of verbal harassment* (direct sexual phrases – Commentary on their body, look, clothes, or lifestyle).
- *Four types of non-verbal harassment* (sexually suggestive looks or gestures – exposure to printed photos with sexual content- exposure to a direct indecent appearance – being followed closely by someone)
- *Three types of physical harassment* (inappropriate touches – an attempt of rape – complete rape).
- *Four types of cyber-harassment* (exposure to sexual photos or videos without their consent – exposure to online blackmail to do online sexual acts – exposure to bullying using sexual misogynistic phrases – receiving requests to perform sexual acts without proper context and consent).
- They were also asked about the *frequency of getting harassed* (once daily – once monthly, once weekly – a few times in their lifetime).

The questions were reproduced from Fitzgerald et al. [23]. They were revised for comprehensiveness, clarity, and relevance (face validity) by the public health and community medicine staff at the faculty of medicine of Alexandria University.

2. *The second part* asked yes/No questions about the setting of harassment (work/ school or college/ public transport/ street or public place/home), the type of harasser (A friend/ a colleague/ a teacher, boss or power holder/ a family member/ a stranger), the feelings associated with the situation (fear/ embarrassment/ anger or desire to revenge/ defeat/neutral feeling), and the type of support given if any (Friends/family/ counseling service/police or governmental office). The public health and community medicine staff at faculty of medicine at Alexandria University revised the questions.

A full Arabic and English version of the questionnaire can be found in Appendix.1

### Sampling and sample size calculation

We used convenience sampling to acquire the participants’ responses via online survey distribution. An Independent sample size was calculated for each age group in each country separately using the equation *n* = z^2^P(1−P)/d^2^ [25]. Under a 95% CI 50% response distribution, and 0.05 margin of error, a sample of 384 participants can be considered a minimal sample representing big populations. However, due to the limitations of convenience sampling and online surveying of the potential participants, we included a design effect (DE) factor in the equation. DE is the ratio of the variance of the estimate observed with a certain type of sampling to the expected variance of the estimate had the sample been collected using simple random sampling (SRS).it was recommended to be 2 to 4 by some Studies [26]. So we used a design effect of 2 to 4 multiplied by the minimal sample size calculated by the previous equation as a correction factor to adjust the sample size. Finally, a minimum sample of 768 to 1536 participants was considered for sampling each age group (18–25, 25–30, 30–35) in each country.

### Ethical considerations

The ethical approval was obtained from the IRB committee in the Faculty of Medicine, Alexandria University, Egypt, on 15th October 2020, under serial number 0304838. And all the methods were performed according to the relevant guidelines and regulations. All the participants gave informed consent that they agreed to complete the questionnaire for research purposes.

### Statistical analysis

- Descriptive statistics (Frequency and percentages) were used to describe the participants’ demographic characteristics and the prevalence of both PMS and PMDD after properly scoring the responses to the screening tool.
- The Chi-square test was used to compare the frequency of PMS and PMDD according to the different demographic characteristics of the participants.
- For the Assessment of the association with harassment, a score was developed to assess “the diversity of the harassment experience” of the participants by giving a score of 1 or 0 to each type of harassment according to whether or not it was experienced by the participant yielding a score range from 0 to 13 for each participant. The Shapiro-Wilk test tested the normality of the score, and accordingly, the Mann-Whitney test was used to compare the scores between those who had PMS/PMDD and those who did not. In addition, chi-square analysis was used to compare the frequencies of PMS and PMDD concerning the frequency of harassment, types of harassers reported, the setting of harassment, feelings encountered, and who presented support.

## Results

### Demographic characters of the study sample

The total study sample was 22,021 female participants from the three included countries. 46.6% of them were from Egypt, 21.2% from Jordan and 32.2% from Syria.

Most of the study sample (65.1%) aged 18–25 years old. 75.5% had a college degree and 75.9% were single or separated. See Table 1 for full details.

**Table 1:** Demographic characters of the study sample (*n* = 22,021)

Basic characteristics	Egypt (*n* = 10,264)	Jordan (*n* = 4665)	Syria (*n* = 7092)	Total n (%)
Age				
18-25 years 25-30 years 30-35 years *BMI* Underweight Normal Over-weight Obese	6531 2189 1544 279 4935 3301 1749	2808 1015 842 227 2962 1104 372	4996 1366 730 585 4926 1271 310	**14,335 (65.1%)** **4570 (20.8%)** **3116 (14.2%)** **1091 (5%)** **12,823 (58.2%)** **5676 (25.8%)** **2431 (11%)**
Work				
Employed Unemployed Student	5069 1219 3976	1825 1334 1506	2544 1197 3351	**9438 (42.9%)** **3750 (17.0%)** **8833 (40.1%)**
Education level				
Below College College degree above college degree	871 7525 1868	894 3380 391	773 5725 594	**2538 (11.5%)** **16,630 (75.5%)** **2853 (13%)**
Marital status				
Single or separated married widow/divorced	7396 2713 156	3349 1250 66	5652 1339 101	**16,397 (75.9%)** **5302 (24.1%)** **323 (1.5%)**

### Prevalence of Premenstrual syndrome (PMS) and Premenstrual dysphoric disorder (PMDD) among the study participants

Amongst the total 22,021 participants, a total number of 16,079 fulfilled the criteria for premenstrual syndrome (73%), whereas 7727 fulfilled the PMDD criteria (35%).

### Relationship between PMS and PMDD prevalence and the participants' demographic characters

The frequency of PMS was significantly higher among obese and overweight participants than normal and underweight participants (*P* = 0.0001), also the prevalence of PMS was significantly higher among participants from Egypt (77.7%), followed by those from Jordan (72.9%) then Syrians (66.3%). Table 2.

**Table 2:** Association between the frequency of PMS among the participants and their demographics (Chi-square test)

	Have PMS (*n* = 16,079)	PMS Free (*n* = 5942)	***P*** value
Age			.26
18–25 years	10,408 (72.6%)	3927 (27.4%)	
25–30 years	3401 (74.4%)	1169 (25.6%)	
30–35 years	2270 (72.8%)	846 (27.2%)	
BMI			.000*
Underweight	776 (71.1%)	315 (28.9%)	
Normal	9271 (72.3%)	3552 (27.7%)	
Over-weight	4195 (73.9%)	1481 (26.1%)	
Obese	1837 (75.6%)	594 (24.4%)	
Country			.000*
Egypt	7977 (77.7%)	2287 (22.3%)	
Jordan	3400 (72.9%)	1265 (27.1%)	
Syria	4702 (66.3%)	2390 (33.7%)	
Education level			.002*
Below College	1814 (71.5%)	724 (28.5%)	
College degree	12,118 (72.9%)	4512 (27.1%)	
above college degree	2147 (75.3%)	706 (24.7%)	
Marital status			.46
Single or separated	12,187 (72.9%)	4532 (27.1%)	
married	3892 (73.4%)	1410 (26.6%)	
Job			.000*
Medical Job	4502 (76.1%)	1415 (23.9%)	
Office Job	1131 (71.9%)	442 (28.1%)	
Service Job	1211 (70.8%)	499 (29.2%)	
Business owner	171 (71.8%)	67 (28.2%)	
Student	6388 (72.3%)	2445 (27.7%)	
Never worked before	2676 (71.4%)	1074 (28.6%)	
Number of work hours			.16
Less than 20 h	3906 (73.7%)	1397 (26.3%)	
20–40 h	6215 (73%)	2298 (27%)	
40–60 h	1152 (73.8%)	409 (26.2%)	
More than 60	244 (68.9%)	110 (31.3%)	
No current job	4562 (72.5%)	1728 (27.5%)	
Number of Pregnancies**			.76
Zero	965 (72.7%)	363 (27.3%)	
1	1150 (74.8%)	387 (25.2%)	
2	1131 (72.4%)	431 (27.6%)	
Three or more	873 (74.2%)	303 (25.8%)	
Number of births**			.16
Zero	1262 (71.9%)	494 (28.1%)	
1	1158 (74.9%)	389 (25.1%)	
2	1124 (73.3%)	409 (26.7%)	
Three or more	558 (75%)	186 (25%)	

The * indicates statistical significance, ** the number of pregnancies is reported for 5603 participants and the number of births is reported for 5580 participants

Participants with higher educational levels had significantly higher PMS prevalence, and those who work in the medical field had a higher prevalence than other professions. Age, marital status, and number of hours spent working did not significantly affect the frequency of PMS. Table 2.

The Frequency of PMDD was also higher among those with higher body weight (the overweight and obese) than those who are normal or underweight. The prevalence was significantly higher among Egyptians (40%), followed by Jordanians (34.7%) then Syrians (28.2%). Table 3.

**Table 3:** Association between the frequency of PMDD among the participants and their demographics (Chi-square test)

	Have PMDD (*n* = 7727)	PMDD Free (*n* = 14,294)	*P* value
Age			.9
18–25 years	5006 (34.9%)	9329 (65.1%)	
25–30 years	1646 (36%)	2924 (64%)	
30–35 years	1075 (34.5%)	2041 (65.5%)	
BMI			.001*
Underweight	372 (34.1%)	719 (65.9%)	
Normal	4363 (34%)	8460 (66%)	
Over-weight	2121 (37.4%)	3555 (62.6%)	
Obese	871 (35.8%)	1560 (64.2%)	
Country			.000*
Egypt	4106 (40%)	6158 (60%)	
Jordan	1620 (34.7%)	3045 (65.3%)	
Syria	2001 (28.2%)	5091 (71.8%)	
Education level			.24
Below College	874 (34.4%)	1664 (65.6%)	
College degree	5827 (35%)	10,803 (65%)	
above college degree	1026 (36%)	1827 (64%)	
Marital status			.8
Single or separated	5859 (35%)	10,860 (65%)	
married	1868 (35.2%)	3434 (64.8%)	
Job			.000*
Medical Job	2271 (38.4%)	3646 (61.6%)	
Office Job	484 (30.8%)	1089 (69.2%)	
Service Job	578 (33.8%)	1132 (66.2%)	
Business owner	83 (34.9%)	155 (65.1%)	
Student	3087 (34.9%)	5746 (65.1%)	
Never worked before	1224 (32.6%)	2526 (67.4%)	
Number of work hours			
Less than 20 h	1879 (35.4%)	3424 (64.6%)	.371
20–40 h	3004 (35.3%)	5509 (64.7%)	
40–60 h	547 (35%)	1014 (65%)	
More than 60	101 (28.5%)	253 (71.5%)	
No current job	2196 (34.9%)	4094 (65.1%)	
Number of Pregnancies**			.8
Zero	466 (35.1%)	862 (64.9%)	
1	552 (35.9%)	985 (64.1%)	
2	549 (35.1%)	1013 (64.9%)	
Three or more	422 (35.9%)	754 (64.1%)	
Number of births**			.19
Zero	598 (34.1%)	1158 (65.9%)	
1	552 (35.7%)	995 (64.3%)	
2	563 (36.7%)	970 (63.3%)	
Three or more	266 (35.8%)	478 (64.2%)	

The * indicates statistical significance, ** the number of pregnancies is reported for 5603 participants and the number of births is reported for 5580 participants

The Age, Education level, marital status and number of hours of work did not significantly affect the PMDD frequency, whereas those who worked in a medical job showed a higher frequency of PMDD than other professions. Table 3.

### Relationship between PMS, PMDD and harassment among the study participants

A total number of 5733 participants reported that they were harassed before and consented to fill the part of the questionnaire assessing harassment. The prevalence of PMS among this sample was 78.9% and the prevalence of PMDD was 44.3%. The score reflecting the diversity of the harassment experience was significantly higher among those who fulfilled the criteria for PMS and PMDD than those who did not. Table 4.

**Table 4:** Relationship between PMS and PMDD and the "Harassment experience" score (Mann–Whitney test), (n = 5733)

	PMS		*p*	PMDD		*p*
	Have PMS	PMS free		Have PMDD	PMDD free	
Harassment experience score	4.4 (2.2)	3.8 (2.1)	.000*	4.6 (2.3)	4.0 (2.1)	.000*

Data presented as Mean (*SD*), * indicates significance

Also, the frequency of having PMS was higher in those who reported being harassed on daily basis (87.6%) than those who were harassed once weekly or monthly (80.7%) and those harassed few times in their lifetime (78%), the differences were statistically significant (*P* = 0.004). A similar statistically significant difference was noticed regarding having PMDD (66.4% VS 47.6% VS 42.5%).

Regarding the setting of harassment, being harassed at School/college or public transportation was significantly associated with a higher frequency of both PMS and PMDD among the women who have been harassed, while being harassed at home did not.

Regarding the type of harasser, being harassed by a family member was significantly associated with higher frequency of both PMS and PMDD. And being harassed by a friend or a colleague was associated with higher frequency of PMDD.

Regarding the feelings felt during harassment, reporting a feeling of fear, embarrassment, Anger/desire to revenge, or defeat was significantly associated with higher frequency of both PMS and PMDD. Reporting feeling neutral did not reach such significant association.

Also, women who reported receiving support from a professional counseling service showed significantly higher levels of having PMS and PMDD, while those who were supported by their families or the police did not. See Additional file 1: Supplementary File 1 for full details.

## Discussion

Our results indicate a very high prevalence of Premenstrual disorder among women from the included Arab countries, which is much higher than the general prevalence (77.7%, 72.95, 66.3% Vs 47.8%). This is the first study to target a considerable sample with multiple age groups. This study also sheds light on a potential association between sexual harassment and PMS. This association was first proposed in a study by Romito et al.[15]. However, it is immediately unclear how sexual harassment might lead to pre-menstrual disorders.

Due to the difference in the methods of collecting data and Criteria for diagnosing the condition, there is a wide difference in the previously published data from the included countries. Most of the Egyptian published data were from adolescent or very young females at secondary schools. The largest study reported a PMS prevalence of 65% [17]. A study on Jordanian university students and workers reported PMS and PMDD prevalence of 80.2% and 10.2 respectively [20].

The large prevalence we found in our study may carry an overestimation of the actual prevalence. Studies showed that different factors affect symptom reporting rates among patients. Paying attention to the symptom and long-term experience of the disease showed the greatest effect. Also longer recall periods may share in overestimation [29]. All of these were particularly observed in our study. In the last decade, there has been a great change in the Arab cultures regarding discussing normal physiological female body changes whether during PMS or pregnancy and postpartum. Women have become more aware of their own bodies. PMS and other sexual topics have been brought up on public platforms like social media. This has made more women primed to report symptoms that they hadn’t recognized before. This is accentuated by the fact that the largest group of participants in our study is highly educated.

In line with our results, a significant association was established between the medical profession and PMS prevalence. Among medical students, 35.6% of Saudi girls [30], 39.4% of Iranian girls [31], and 51% of Pakistani girls [32] reported PMS symptoms. In fact, higher educational levels, in general, are associated with higher PMS prevalence [33, 34]. Women who engage in a more professional environment showed more recognition and perception of their own symptoms, and thus higher reporting rate. Both associations could be accounted for by the increased level of stress, which is the main risk factor for PMS. The severity of the premenstrual symptoms in each cycle is associated with the level of stress during the previous month [35]. Women exposed to high stress were 25 times more likely to report severe symptoms than those who weren't exposed [35]. And in this aspect, we were Concerned about Data collection timing During the Covid-19 Pandemic and after a long period of lockdown. Studies showed that psychological symptoms are triggered during outbreak times causing an increase in the severity of symptoms [36]. These effects were observed in both people with previous pathologies and healthy individuals and thus it may have affected the reported numbers of disease prevalence [37].

The association between obesity and PMS prevalence we observed in our results was also documented. With each 1 kg/m2 increase in BMI, PMS risk increases by 3% [38]. That was best explained by the effect of gonadal hormones on the serotonin level, which is involved in PMS symptoms development [39, 40]. The type of diet also showed to have a strong influence on symptom reporting. Symptoms increased in women with high caloric food intake that contains high amounts of fat, sugar, and salt, while greatly decreased with increased healthy food intake like fruit [41].

Although we found no significant association between age of the patients and prevalence of PMS, several studies confirmed this association. Bianco et al. found that PMS is more common the young age group [42]. Similarly, Tarannum et al. stated that PMS was 2.9 times more common in the late adolescence (15–19 years) [43]. Another cross-sectional study by farahmand et al. disputes with our results and suggests a significant correlation between age of the patients and their marital status and increased severity of PMS symptoms[44]. Marital status was also found to affect PMS symptoms severity in another study on Jordanian women by Hamaideh et al. [20].

This study provided data about the association between Sexual harassment and the prevalence of premenstrual disorders. Trauma following sexual harassment (SH) is associated with higher post-traumatic stress symptoms, depressive symptoms, and suicidal attempts [45]. Sexual Harassment has become a trending issue in the Arab region in the last decade; a national survey found that 40% of Egyptian young women reported SH in 2014. In a more recent report that involved 12 Arab countries, Egypt ranked first in both Verbal and physical harassment with 42% and 29% of the population sample respectively while Jordanian women reported 21% and 6% in the two aspects [46]. It is unclear why increased sexual harassment experience is associated with increased levels of premenstrual disorders, but this study sheds light on a potential link that needs to be further investigated. This study also provided data on the association between premenstrual disorders and the setting of harassment, type of perpetrator, feelings encountered and type of support presented. These reported associations can be helpful in providing some sort of insight about how the specifications of the harassment experience might lead to development of premenstrual disorders.

### Strengths and limitations of the study

Strength points of the study include reporting data about a sensitive topic from an under-studied population as the Arab region, in addition to the large sample size and diverse demographics of the study sample. Main limitations are the use of convenience online based sampling, and only recruiting women within the 18–35 age range, these limitations stand against the generalization of the study results.

## Conclusion

In conclusion, PMS and PMDD are highly prevalent conditions among Arab women according to this study sample. The conditions are more prevalent in Egypt than Jordan and Syria. There is no significant association between the prevalence of the condition and Age, marital status or Number of work hours, while it’s associated with multiple factors such as body mass index, higher educational levels, and working in medical professions. Sexual harassment is suggested to be linked to pre-menstrual disorders but further work is still needed to establish such association and track its dynamics. Our results are substantial to the raise the awareness of PMS and PMDD in the clinical practice, and to provide the basis for future research to investigate the prevalence of PMS and PMDD and prove the association with SH in a larger samples including more age groups.

## Supplementary Information

**Additional file 2. Supplementary table (1):** Relationship between PMS and PMDD frequency in the people who reported harassment and the type of harasser and setting of harassment.

## Data Availability

All data are available from the corresponding author upon reasonable request.
