# Supplementary material for: Prevalence and patterns of premenstrual disorders and possible association with sexual harassment: a cross-sectional study of young Arab women
Source: BMC Womens Health. 2022 Dec 21;22:536. doi: 10.1186/s12905-022-02130-0 (PMC9768784; doi:10.1186/s12905-022-02130-0)
Supplement: Supplementary file 1 — Additional file 2. Supplementary table (1): Relationship between PMS and PMDD frequency in the people who reported harassment and the type of harasser and setting of harassment. [file 12905_2022_2130_MOESM1_ESM.docx]

Supplementary table (1): Relationship between PMS and PMDD frequency in the people who reported harassment and the type of harasser and setting of harassment. (n= 5733)

|  | Have PMS (n=4522) | PMS free (n= 1211) | Sig | Have PMDD (n=2538) | PMDD free (n=3195) | Sig |
| --- | --- | --- | --- | --- | --- | --- |
| Setting of harassment | | | | | | |
| **Work**   - Yes - No | 295 (82.4%)  4227 (78.6%) | 63 (17.6%)  1148 (21.4%) | .09 | 184 (51.4%)  2354 (43.8%) | 174 (48.6%)  3021 (56.2%) | .005* |
| **School/college**   - Yes - No | 497 (82.7%)  4025 (78.4%) | 104 (17.3%)  1107 (21.6%) | .015* | 316 (52.6%)  2222 (43.3%) | 285 (47.4%)  2910 (56.7%) | .000* |
| **Public transport**   - Yes - No | 2258 (81.5%)  2264 (76.5%) | 514 (18.5%)  697 (23.5%) | .000* | 1330 (48%)  1208 (40.8%) | 1442 (52%)  1753 (59.2%) | .000* |
| **Street/public places****   - Yes - No | 2685 (80.2%)  1608 (76.5%) | 661 (19.8%)  495 (23.5%) | .001* | 1509 (45.1%)  904 (43%) | 1837 (54.9%)  1199 (57%) | .13 |
| **Home**   - Yes - No | 591 (81.3%)  3931 (78.5%) | 136 (18.7%)  1075 (21.5%) | .09 | 341 (46.9%)  2197 (43.7%) | 386 (53.1%)  2809 (56.1%) | .13 |
| Type of harasser | | | | | | |
| **A friend**   - Yes - No | 264 (81.5%)  4258 (78.7%) | 60 (18.5%)  1151 (21.3%) | .24 | 173 (53.4%)  2365 (43.7%) | 151 (46.6%)  3044 (56.3%) | .001* |
| **A colleague**   - Yes - No | 354 (82.1%)  4168 (78.6%) | 77 (17.9%)  1134 (21.4%) | .09 | 221 (51.3%)  2317 (43.7%) | 210 (48.7%)  2985 (56.3%) | .002* |
| **A teacher /boss/ power holder****   - Yes - No | 68 (70.1%)  4096 (78.5%) | 29 (29.9%)  1125 (21.5%) | .05 | 45 (46.4%)  2265 (43.4%) | 52 (53.6%)  2956 (56.6%) | .55 |
| **A family member**   - Yes - No | 851 (81.3%)  3671 (78.3%) | 196 (18.7%)  1015 (21.7%) | .035* | 501 (47.9%)  2037 (43.5%) | 546 (52.1%)  2649 (56.5%) | .01* |
| **A stranger**   - Yes - No | 3430 (79.9%)  1092 (75.8%) | 863 (20.1%)  348 (24.2%) | .001* | 1909 (44.5%)  629 (43.7%) | 2384 (55.5%)  811 (56.3%) | .6 |

The * indicates significance, ** the street/public place response is present for 5499 participants, and for a teacher/boss/power holder is present for 5318 participants.

Supplementary table (2): Relationship between PMS and PMDD frequency in the people who reported harassment and the type of support and the feeling associated. (n=5733)

|  | Have PMS (n=4522) | PMS free (n= 1211) | Sig | Have PMDD (n=2538) | PMDD free (n=3195) | Sig |
| --- | --- | --- | --- | --- | --- | --- |
| Feelings associated | | | | | | |
| **Fear**   - Yes - No | 2113 (82.7%)  2409 (75.8%) | 443 (17.3%)  768 (24.2%) | .000* | 1197 (46.8%)  1341 (42.2%) | 1359 (53.2%)  1836 (57.8%) | .000* |
| **Embarrassment**   - Yes - No | 993 (82.3%)  3529 (78%) | 213 (17.7%)  998 (22%) | .001* | 566 (46.9%)  1972 (43.6%) | 640 (53.1%)  2555 (56.4%) | .036* |
| **Anger/ desire to revenge**   - Yes - No | 2947 (80.8%)  1575 (75.5%) | 699 (19.2%)  512 (24.5%) | .000* | 1669 (45.8%)  869 (41.6%) | 1977 (54.2%)  1218 (58.4%) | .002* |
| **Defeat**   - Yes - No | 1118 (84.4%)  3404 (77.2%) | 206 (15.6%)  1005 (22.8%) | .000* | 691 (52.2%)  1847 (41.9%) | 633 (47.8%)  2562 (58.1%) | .000* |
| **Neutral feeling**   - Yes - No | 591 (77.8%)  3931 (79%) | 169 (22.2%)  1042 (21%) | .42 | 331 (43.6%)  2207 (44.4%) | 429 (56.4%)  2766 (55.6%) | .67 |
| Type of support in response to harassment (Who supported you) | | | | | | |
| **Friends**   - Yes - No | 701 (82.1%)  3821 (78.3%) | 153 (17.9%)  1058 (21.7%) | .01* | 387 (45.3%)  2151 (44.1%) | 467 (54.7%)  2728 (55.9%) | .5 |
| **Family**   - Yes - No | 749 (79.8%)  3773 (78.7%) | 190 (20.2%)  1021 (21.3%) | .47 | 397 (42.3%)  2141 (44.7%) | 542 (57.7%)  2653 (55.3%) | .18 |
| **Counseling service**   - Yes - No | 121 (87.1%)  4401 (78.7%) | 18 (12.9%)  1193 (21.3%) | .02* | 76 (54.7%)  2462 (44%) | 63 (45.3%)  3132 (56%) | .01* |
| **Police or a governmental office**   - Yes - No | 33 (86.8%)  4489 (78.8%) | 5 (13.2%)  1206 (21.2%) | .23 | 18 (47.4%)  2520 (44.2%) | 20 (52.6%)  3175 (55.8%) | .7 |

The * indicates significance.
